# Cross-species metabolic profiling of follicular compartments in obesity

**DOI:** 10.1093/lifemedi/lnag028

**Published:** 2026-07-11

**Authors:** Yiqiu Wu, Shiyu Xie, Mengyuan Zhu, Yu Tian, Hengjie Wang, Shoubin Tang, Minjian Chen, Qiang Wang, Longsen Han, Shuai Zhu

**Affiliations:** State Key Laboratory of Reproductive Medicine and Offspring Health, Nanjing Medical University, Nanjing 211166, China; State Key Laboratory of Reproductive Medicine and Offspring Health, Nanjing Medical University, Nanjing 211166, China; Key Laboratory of Modern Toxicology of Ministry of Education, School of Public Health, Nanjing Medical University, Nanjing 211166, China; State Key Laboratory of Reproductive Medicine and Offspring Health, Center for Reproductive Medicine, Institute of Women, Children and Reproductive Health, Shandong University, Jinan 250012, China; Nanjing Women and Children’s Healthcare Hospital, Women’s Hospital of Nanjing Medical University, Nanjing 210004, China; State Key Laboratory of Reproductive Medicine and Offspring Health, Nanjing Medical University, Nanjing 211166, China; Key Laboratory of Modern Toxicology of Ministry of Education, School of Public Health, Nanjing Medical University, Nanjing 211166, China; State Key Laboratory of Reproductive Medicine and Offspring Health, Nanjing Medical University, Nanjing 211166, China; Changzhou Maternity and Child Health Care Hospital, Changzhou Medical Center, Nanjing Medical University, Changzhou 213000, China; State Key Laboratory of Reproductive Medicine and Offspring Health, Nanjing Medical University, Nanjing 211166, China; State Key Laboratory of Reproductive Medicine and Offspring Health, Nanjing Medical University, Nanjing 211166, China

**Keywords:** obesity, metabolic profiling, granulosa cells, follicular microenvironment

## Abstract

Impaired fertility in obese women is closely associated with disruptions in follicular microenvironment homeostasis. However, the underlying metabolic signatures remain poorly characterized. In this study, we employed a high-fat diet induced obese mouse model and conducted comparative metabolomic analyses of granulosa cells, follicular fluid, and serum from obese mice and women. Our results revealed prevalent metabolic disturbances across species, including dysregulated amino acid metabolism, suppressed steroidogenesis, and impaired fatty acid β-oxidation. Notably, ceramide accumulation in granulosa cells and follicular fluid suggested an insulin-resistant follicular microenvironment. These findings indicate obesity-induced metabolic reprogramming in the follicular niche, highlighting conserved disruptions in energy metabolism and steroidogenic capacity. Overall, our data provide a metabolic framework to elucidate obesity-associated subfertility and suggest potential metabolic targets for diagnosing and treating reproductive disorders.

## Introduction

In the mammalian ovary, oocytes undergo development within follicles, structures composed of granulosa cells (GCs), theca cells, the basal lamina, and follicular fluid (FF). GCs play a pivotal role in follicular development by converting androgens to estrogens and synthesizing progesterone [1]. Functioning as the primary somatic support cells within the follicle, GCs supply essential nutrients crucial for the growth and maturation of oocytes [2]. In addition, GCs and oocytes can exchange substances and signals through gap junctions, and their interactions regulate follicle maturation, ovulation, and fertilization [3]. Previous studies have focused on identifying factors secreted by oocytes and GCs, which serve as biomarkers for oocyte quality, maturation, fertilization potential, embryo development, and GC functionality. Metabolic disorders, particularly obesity, have been shown to cause meiotic abnormalities in oocytes and impair ovulation, leading to subfertility or infertility [4, 5]. Emerging evidence suggests that the metabolic crosstalk between GCs and oocytes has been proposed as a key factor influencing these processes [6–9]. FF originates from blood that flows through the thecal capillaries. During the oocyte maturation, the local microenvironment, especially FF, likely influences gamete quality [10]. Moreover, serum, as a source of systemic metabolites, may also have a significant impact on the metabolic homeostasis of the follicle and the quality of the oocyte. Therefore, as key components of the follicular metabolic microenvironment, identifying the metabolic changes in GCs, FF, and serum can provide new biomarkers for assessing oocyte quality and offer potential intervention targets for improving the success rates of assisted reproductive technologies.

Obesity has become a global health challenge, significantly impacting reproductive health and fertility. It has been observed to be linked with altered risks of numerous female reproductive conditions including polycystic ovary syndrome, abnormal uterine bleeding, endometriosis, infertility, and pregnancy-related disorders. Furthermore, obesity has been shown to significantly prolong the time to conception in women [11–13]. Given the adverse effects of obesity on reproductive health, further research is needed to elucidate the underlying mechanisms and improve reproductive outcomes.

While previous studies have characterized the fundamental metabolic features of mouse oocytes and GCs under physiological conditions, the specific metabolic reprogramming of the follicular environment induced by obesity remains poorly understood. Moreover, existing studies have predominantly focused on individual species or isolated follicular compartments, leaving a gap in our understanding of how obesity systematically orchestrates metabolic alterations across multiple follicular compartments. In this study, we employed ultra-high-performance liquid chromatography (UHPLC)–tandem mass spectrometry to obtain the metabolic profiles of GCs from obese mice and the comprehensive metabolic profiles of the human follicular compartments, including GCs, FF, and serum. Through comparative analysis, we identified both species-specific and conserved metabolic alterations induced by obesity. These findings help elucidate metabolic perturbations in the follicular microenvironment following maternal obesity exposure and provide novel insights into oocyte microenvironmental changes. Furthermore, this work establishes a crucial scientific foundation for developing diagnostic tools and intervention strategies for obesity-related reproductive disorders.

## Results

### Metabolomic profiling of granulosa cells from high-fat diet and normal diet mice

**Figure 1.**
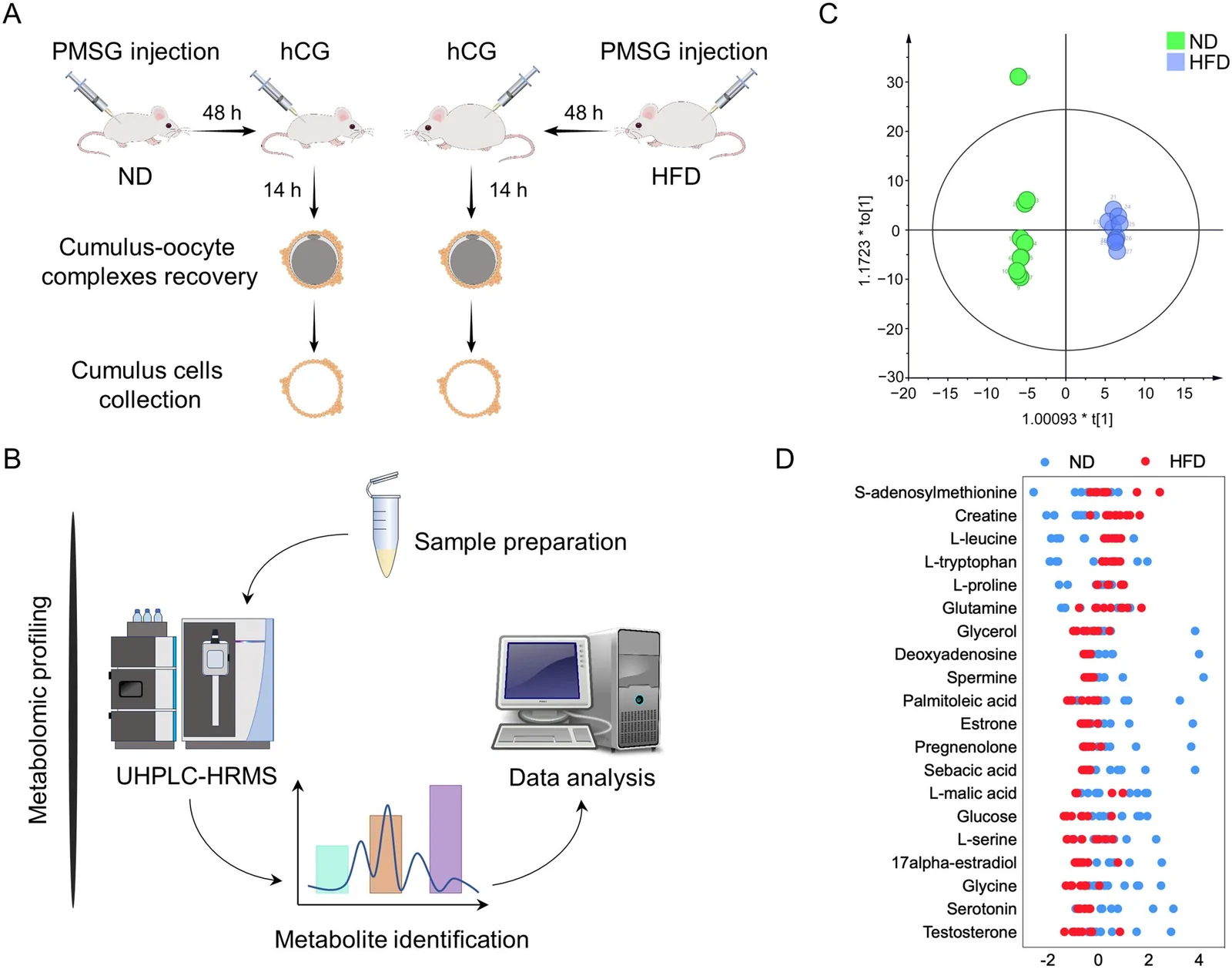
Metabolomic profiling of mouse granulosa cells. (A) Illustration of *in vivo* isolation of mouse GCs. (B) Workflow for UHPLC–HRMS-based metabolome profiling on mouse GCs. (C) OPLS-DA score plot for metabolomic datasets clearly distinguishes GC samples from two groups. (D) *Z*-score plots of 20 representative differential metabolites. The number of biological replicates is *n* = 10 for the ND group and *n* = 9 for the HFD group. ND, normal diet; HFD, high-fat diet.

Granulosa cells surrounding oocytes serve essential functions in oocyte nutrition and mediate communication between the oocyte and its extracellular microenvironment. To investigate obesity’s impact on GC metabolism, we established high-fat diet (HFD) induced obese mouse model. Here, we collected GCs surrounding MII oocytes from the ampulla of oviducts post hormonal stimulation (Fig. 1A), and analyzed the intracellular metabolome using UHPLC high-resolution mass spectrometry (UHPLC–HRMS) (Fig. 1B). Orthogonal partial least squares-discriminant analysis (OPLS-DA) clearly separated the normal diet (ND) and HFD groups, with the cross validated predictive ability of Q2 (cum) ≥ 0.772 (Fig. 1C). A total of 71 differential metabolites were identified through the combination of Student’s *t*-test (*P *< 0.05) and variable importance in projection (VIP > 1.00) analysis (Table S1). Pathway mapping using the Kyoto Encyclopedia of Genes and Genomes (KEGG) revealed distinct biochemical pathways associated with the differential metabolites (Fig. S1A). Twenty representative metabolites are shown in Fig. 1D. KEGG analysis showed a significant enrichment of categories related to amino acid metabolism (Fig. S1B). Collectively, these results demonstrate that obesity significantly alters the metabolic landscape of mouse GCs, particularly affecting amino acid metabolism pathways.

### Amino acids metabolism of granulosa cells from high-fat diet and normal diet mice

**Figure 2.**
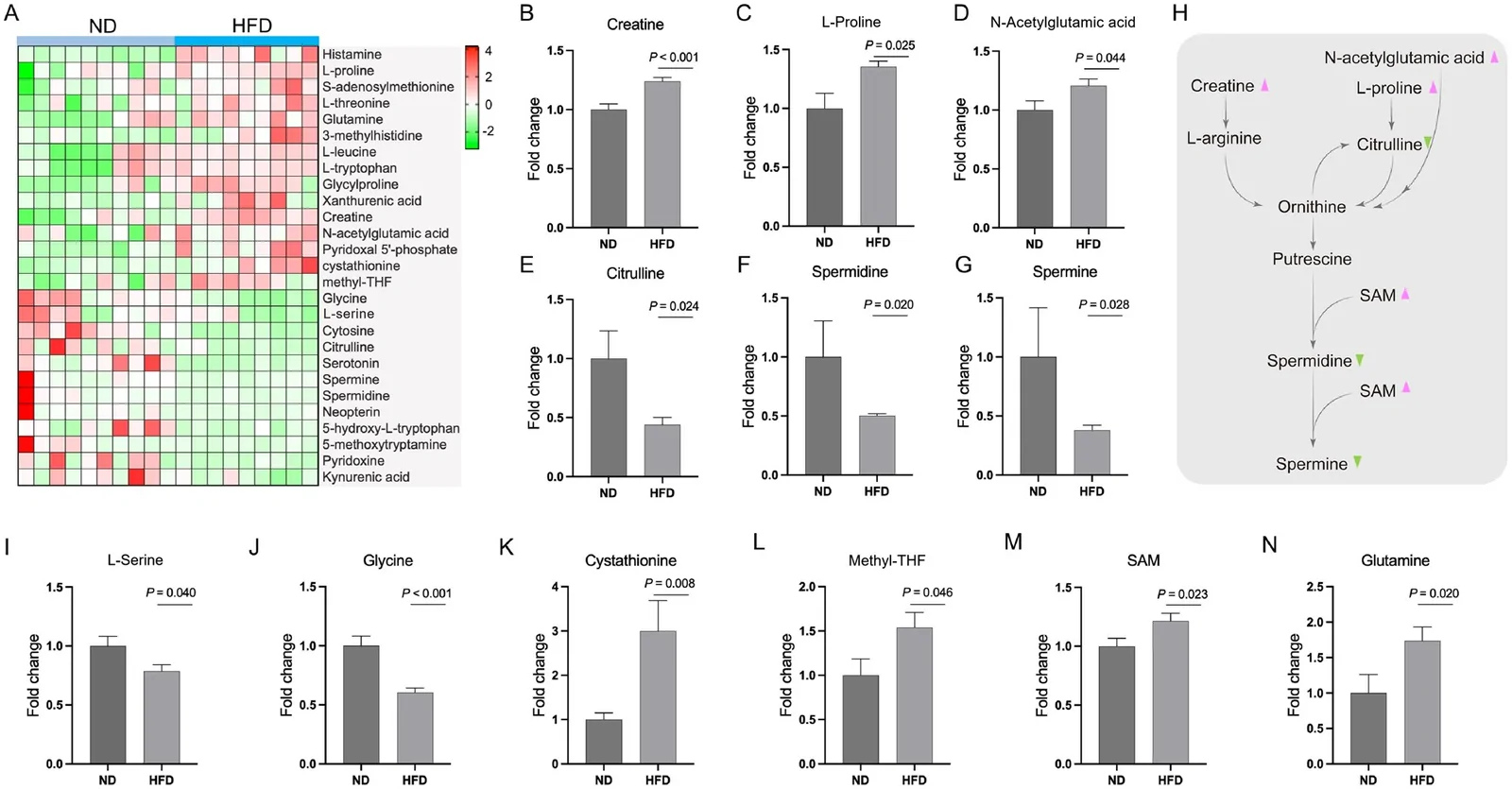
Amino acid metabolism in mouse granulosa cells. (A) Heatmap visualizing the relative abundance of differential amino acid metabolites in GCs from ND and HFD mice. (B–G) Relative levels of metabolites related to polyamine metabolism. (H) Schematic diagram of polyamine metabolism. Metabolites from HFD mice with increased levels are indicated by red triangles, while those with decreased levels are indicated by green triangles. (I–N) Relative levels of metabolites related to SGOC pathway. The number of biological replicates is *n* = 10 for the ND group and *n* = 9 for the HFD group. Error bars, SEM.

Active amino acid metabolism is essential for maintaining GCs homeostasis, supporting oocyte nutrition, and facilitating ovulation [14]. Our metabolite profiling of mouse GCs revealed significant alterations in 27 amino acid metabolites, with 15 showing increased abundance and 12 demonstrating decreased levels (Fig. 2A). Polyamine metabolism, particularly involving spermidine, plays a crucial role in maintaining oocyte quality and female reproductive function by regulating autophagy, mitochondrial function, and antioxidant capacity [15]. The polyamine biosynthesis pathway exhibited distinct metabolic shifts: upstream metabolites (creatine, L-proline, and N-acetylglutamic acid) were upregulated, while downstream products (citrulline, spermine, and spermidine) were downregulated (Fig. 2B–H). The serine-glycine-one-carbon (SGOC) pathway is a critical metabolic network that produces essential metabolites for nucleotide, protein, and lipid biosynthesis and supports epigenetic regulation by fueling methyltransferase reactions [16]. Similarly, the SGOC pathway showed decreased L-serine and glycine levels (Fig. 2I and 2J) but increased cystathionine, methyl-THF, and S-adenosylmethionine (SAM) (Fig. 2K–M). Particularly SAM, which serves as the primary methylation donor for epigenetic modifications [16], may potentially alter the epigenetic modification activity in GCs. Additionally, we detected elevated glutamine levels (Fig. 2N), which previous studies associate with enhanced GCs antioxidant capacity, proliferation, and estrogen (E2) production [17]. These data indicate that HFD disrupts GC amino acid metabolism, with polyamine depletion and SGOC pathway imbalance potentially contributing to oocyte defects, whereas glutamine accumulation may partially mitigate oxidative stress.

### Lipid metabolism of granulosa cells from high-fat diet and normal diet mice

**Figure 3.**
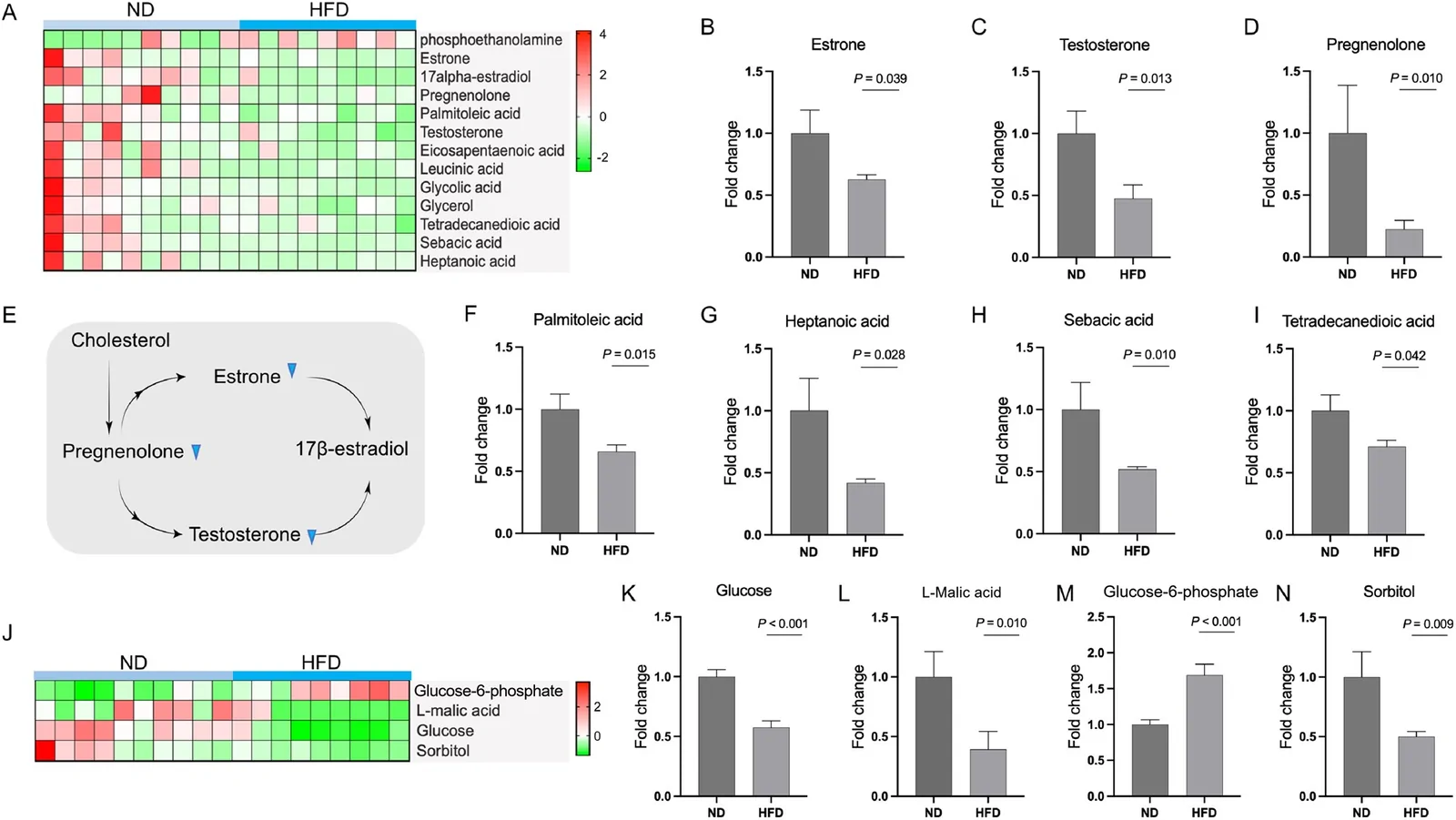
Lipid and carbohydrate metabolism in mouse granulosa cells. (A) Heatmap visualizing the relative abundance of differential lipid metabolites in GCs from ND and HFD mice. (B–D) Relative levels of metabolites related to steroid hormone biosynthesis. (E) Schematic diagram of steroid hormone biosynthesis in mice GCs. Metabolites from HFD with decreased levels are indicated by blue triangles. (F–I) Relative levels of metabolites related to fatty acid β-oxidation. (J) Heatmap visualizing the relative abundance of differential carbohydrate metabolites. (K–N) Relative levels of metabolites related to carbohydrate. The number of biological replicates is *n* = 10 for the ND group and *n* = 9 for the HFD group. Metabolites from HFD mice with decreased levels are indicated by downward-pointing blue triangles. Error bars, SEM.

Beyond amino acid metabolic alterations, our metabolomic analysis identified significant changes in lipid metabolite levels within GCs of HFD mice. Most of these lipid metabolites were downregulated, particularly those involved in steroid hormone biosynthesis and fatty acid metabolism (Fig. 3A). Previous studies have shown that obesity leads to metabolic disorders in steroid hormones, particularly E2 [18, 19]. However, the metabolic profile of steroid hormones in GCs of obese mouse remains poorly characterized. Our data revealed marked reductions in key cholesterol derivatives, including estrone, pregnenolone, and testosterone (Fig. 3B–D). As these compounds serve as essential precursors for E2 biosynthesis (Fig. 3E), their decreased abundance suggests impaired E2 synthesis capacity in GCs under obese conditions.

Fatty acid β-oxidation in GCs provides essential energy for oocyte meiotic maturation via ATP production [20]. In HFD mice, we observed downregulation of palmitoleic acid, heptanoic acid, sebacic acid, and tetradecanedioic acid (Fig. 3F–I). These alterations suggest potential dysregulation in fatty acid metabolism pathways, possibly affecting both lipid catabolism and anabolism. Particularly noteworthy is the reduced abundance of sebacic acid and tetradecanedioic acid, which are direct products of long-chain fatty acid β-oxidation. Collectively, these findings demonstrate that obesity disrupts the lipid metabolic function in GCs, impairing both steroid hormone biosynthesis and fatty acid β-oxidation processes, which may lead to insufficient energy supply for oocytes and diminished hormonal support in the follicular microenvironment.

### Carbohydrate metabolism of granulosa cells from high-fat diet and normal diet mice

Carbohydrates serve as a crucial source of materials for both primary and secondary metabolism [21]. Research indicates that GCs significantly contribute to carbohydrate metabolism. Their key function involves providing glucose and pyruvate to oocytes, which have restricted glucose metabolism capabilities [22]. We found that levels of glucose and L-malic acid were downregulated, while glucose-6-phosphate was upregulated in HFD mice (Fig. 3J–M). Furthermore, sorbitol, a key metabolite of the polyol pathway, supports oocyte maturation by converting to fructose to provide energy substrates and is associated with maintaining oocyte redox balance [23, 24]. Sorbitol levels in HFD GCs were significantly reduced compared to the control group (Fig. 3N). This reduction in energy substrates may disrupt the metabolic support of GCs for oocytes, leading to altered energy supply and increased oxidative stress, thereby impairing oocyte quality and developmental competence.

### Metabolic parallels in granulosa cells between overweight/obese women and mice

**Figure 4.**
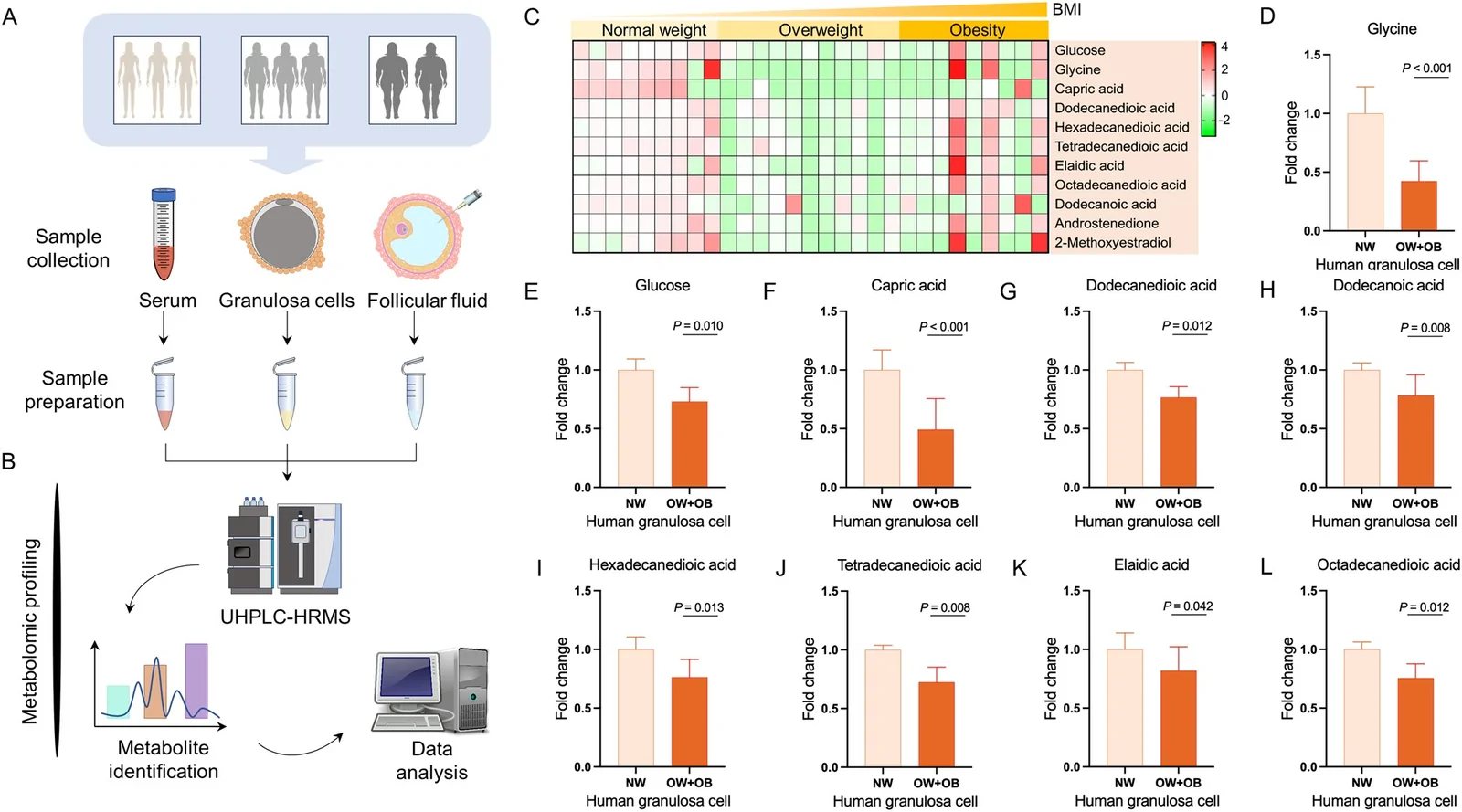
Metabolomic profiling of human granulosa cells. (A) Sample collection and processing workflow for serum, granulosa cells, and follicular fluid from normal-weight (NW), overweight (OW), and obesity (OB) groups. (B) Workflow for UHPLC/HRMS-based metabolome profiling on human samples. (C) Heatmap visualizing the relative abundance of differential metabolites in GCs from NW and OW/OB groups. (D–L) Relative levels of metabolites in GCs from NW and OW/OB groups. The number of biological replicates for granulosa cells is *n* = 9 for the NW group and *n* = 20 for the OW/OB group. Error bars, SEM.

To investigate the metabolic profiles associated with body mass index (BMI) categories, we recruited a total of 45 infertile women with a mean age of 30 ± 5 years (Table S2). Based on BMI classification, participants were divided into two groups: the normal weight (NW) group, with a BMI of 18.5–24.9 kg/m^2^ (*n *= 18); and the overweight/obesity (OW/OB) group, with a BMI ≥ 25 kg/m^2^ (*n *= 27). We collected the following samples from these participants: GC samples from 9 individuals in the NW group and 20 individuals in the OW/OB group; FF samples from 18 individuals in the NW group and 27 individuals in the OW/OB group; and serum samples from 18 individuals in the NW group and 27 individuals in the OW/OB group (Fig. 4A). The metabolome of GCs, as well as the metabolites of FF and serum, were analyzed using UHPLC–HRMS (Fig. 4B and Table S3). OPLS-DA showed clearly separation of the groups in GCs, FF, and serum (Fig. S2A–C).

Our comparative metabolomic analysis revealed conserved metabolic alterations in GCs between HFD mice and OW/OB women. Consistent with our results in mice, KEGG analysis of human GCs revealed significant enrichment in categories related to amino acid metabolism (Fig. S2D). Both species exhibited significant downregulation of key metabolites: glycine (0.60-fold in mice vs. 0.42-fold in humans) and glucose (0.58-fold in mice vs. 0.73-fold in humans) (Figs. 2J, 3K, and 4C–E). Furthermore, we identified consistent downregulation of multiple long-chain fatty acids implicated in β-oxidation pathways, including capric acid, dodecanedioic acid, dodecanoic acid, hexadecanedioic acid, tetradecanedioic acid, elaidic acid, and octadecanedioic acid (Fig. 4F–L). While the specific fatty acid metabolites affected showed partial interspecies variation, the overall suppression of long-chain fatty acid β-oxidation was remarkably conserved between mouse and human (Table S4).

### Metabolic changes in the follicular microenvironment of overweight/obese women

**Figure 5.**
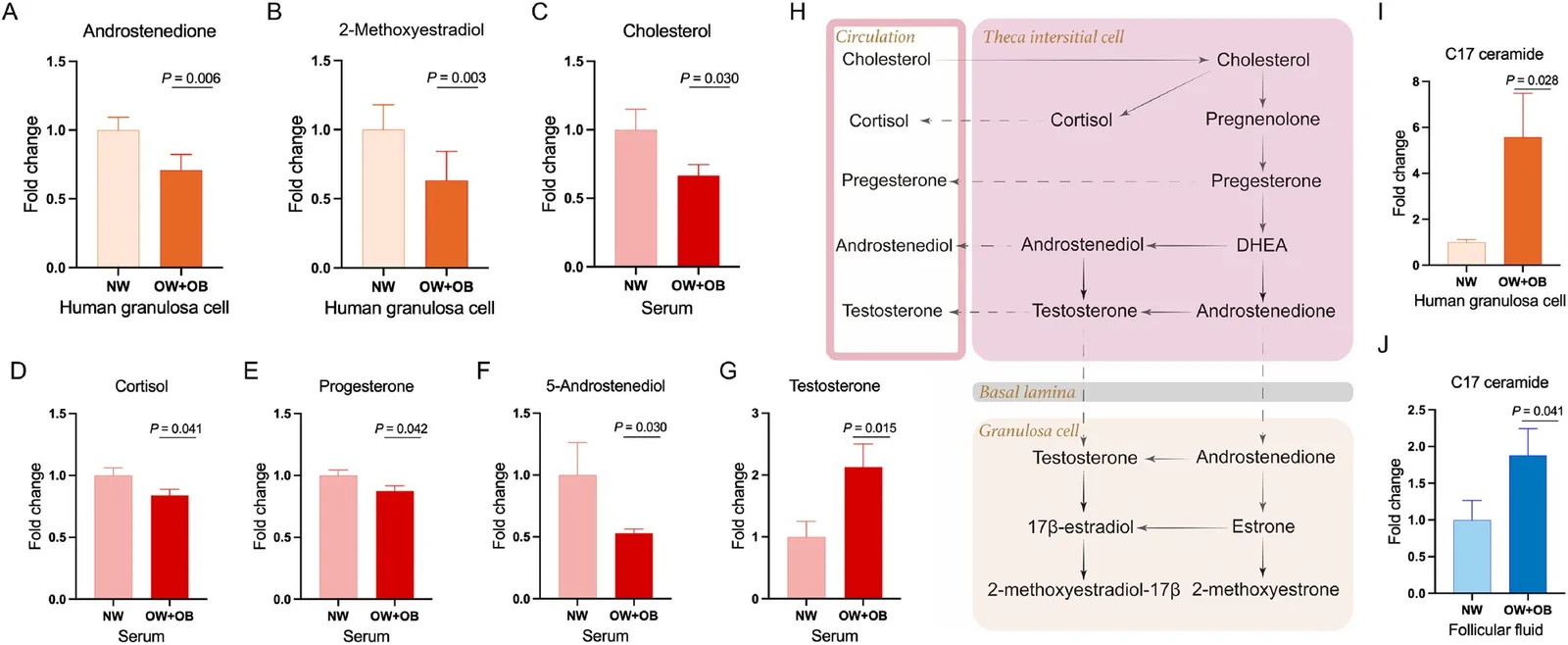
Metabolic profiles in the follicular microenvironment of overweight/obese (OW/OB) women. (A–G) Relative levels of metabolites related to steroid hormone biosynthesis in follicles in NW and OW/OB groups. (H) Schematic diagram of steroid hormone biosynthesis in human follicles. Metabolites from OW/OB with increased levels are indicated by red triangles, while those with decreased levels are indicated by blue triangles. (I, J) Relative levels of C17 ceramide in GCs (I) and FF (J) from NW and OW/OB groups. The number of biological replicates is *n* = 18 for the NW group and *n* = 27 for the OW/OB group in follicular fluid and serum, and *n* = 9 for the NW group and *n* = 20 for the OW/OB group in granulosa cells. Error bars, SEM.

The metabolic microenvironment surrounding oocytes comprises GCs, FF, and serum, collectively influencing oocyte quality and subsequent developmental potential. While previous studies have typically examined individual follicular components, our investigation revealed coordinated metabolic alterations across this microenvironment. GCs play a pivotal role in female reproduction through steroid hormone biosynthesis [25]. According to the “Two-Cell Two-Gonadotropin Theory” of E2 synthesis, theca cells are primarily responsible for synthesizing androgens such as androstenedione, testosterone, and dehydroepiandrosterone. These androgens are then transported to GCs, where they are converted into estrogens, including estradiol and estrone [26]. In OW/OB women, we observed decreased androstenedione and 2-methoxyestradiol levels in GCs, along with reduced cholesterol, cortisol, progesterone, and 5-androstenediol in serum (Fig. 5A–F). Conversely, serum testosterone levels were significantly elevated (Fig. 5G). When considering serum as a proxy for thecal cell activity, these findings suggest overall downregulation of intrafollicular steroidogenesis with a preferential shift toward testosterone production (Fig. 5H). This pattern indicates that obesity-induced metabolic perturbations systemically affect follicular physiology rather than isolated cellular compartments. Remarkably, these changes were similar to the steroid metabolism changes in HFD mice (Fig. 3E), which supports our notion of metabolic parallels between humans and mouse.

Follicular fluid contains metabolites originating from GCs, including ceramide, a key structural component of cell membranes that serves as a precursor for sphingolipid biosynthesis and is modulated by hormonal, metabolic, and inflammatory signals [27]. We observed a significant upregulation of C17-ceramide levels in both GCs (5.6-fold increase) and FF (1.9-fold increase) (Fig. 5I and 5J). Importantly, metabolomic analysis of the matched serum samples revealed a nonsignificant decreasing trend in C17-ceramide levels (Table S3). This distinct compartmental disparity strongly indicates that the elevation of C17-ceramide in FF is not a transudate from the systemic circulation, but rather results from excessive local secretion by GCs. Given that ceramide accumulation induces insulin resistance (IR) in human liver tissue [28], and considering the widespread expression of insulin receptors in ovarian theca cells and GCs [29], our findings suggest that elevated ceramide levels might contribute to an insulin-resistant state within follicles.

## Discussion

This study was designed to determine the metabolic changes in the follicular microenvironment, including GCs and FF composition, related to obesity. Here, we present a comprehensive analysis of the metabolic profiles in HFD mice GCs and human GCs, FF, and serum from OW/OB infertile women. Comparative analysis revealed both conserved and species-specific metabolic signatures across these follicular compartments. Key findings include: (i) dysregulation of amino acid, fatty acid, and carbohydrate metabolism in mouse GCs; (ii) remarkable metabolic parallels between mouse and human GCs; and (iii) coordinated metabolic perturbations throughout the human follicular microenvironment. These findings are the first cross-species comparison of GC metabolic profiles and the most comprehensive characterization of follicular metabolic alterations in obese individuals to date.

In the cumulus-oocyte complex (COC), an extensive network of carbohydrate, amino acid, and lipid metabolic exchanges supports oocyte development and maturation [30–32]. Our data demonstrate that obesity exerts diverse effects on GCs in mouse through metabolic pathways. Reduced antioxidant capacity is observed, characterized by downregulation of glutamine and dysregulation of polyamine metabolism, particularly decreases in spermidine and spermine. Previous studies show that glutamine enhances GC antioxidant capacity by upregulating isocitrate dehydrogenase 1, a marker of oocyte quality [17]. Additionally, *in vitro* supplementation of polyamine metabolites, such as spermine, improves mitophagy and mitochondrial function, reducing reactive oxygen species (ROS) levels and DNA damage in aged mouse. These findings suggest that obesity may impair the antioxidant capacity of GCs by disrupting glutamine and polyamine metabolism.

From an epigenetic standpoint, obesity-induced dysregulation of SGOC metabolism, particularly the elevated SAM, suggests active DNA methylation processes. Interestingly, DNA methylation levels are often elevated in oocytes from obese mouse [33]. This finding suggests a potential mechanistic link between SAM [15] accumulation in GCs and aberrant DNA methylation patterns in oocytes, a hypothesis that merits systematic investigation. Our analysis of hormonal metabolism revealed significant downregulation of E2 biosynthetic precursors within the steroidogenic pathway. While existing literature has established an association between obesity and diminished follicular hormone concentrations [34], the present study clarifies this mechanism. Specifically, the decline is primarily due to decreased secretion of E2 precursors in GCs.

Additionally, our data suggest abnormalities in energy supply within GCs, characterized by reduced glucose levels and decreased fatty acids. Importantly, oocytes have limited capacity for glucose metabolism and rely heavily on GCs for the supply of glucose and pyruvate [22, 35], as their maturation requires highly active pyruvate metabolism and tricarboxylic acid cycle (TCA cycle) activity [16, 36, 37]. Furthermore, long-chain fatty acids, which generate multiple acetyl-CoA molecules through β-oxidation, produce more ATP than glucose. This ATP is subsequently transported from cumulus cells to oocytes, where it plays an indispensable role in maturation processes [38, 39]. In GCs from HFD mouse, reduced long-chain fatty acids impair ATP production from β-oxidation, leading to insufficient energy supply for GCs and further compromising their ability to support oocyte energy requirements and maturation. Collectively, obesity exerts diverse effects on GCs through metabolic pathways, thus impairing oocyte quality and reproductive health.

To date, research on GCs metabolism has predominantly focused on metabolic changes within a single species. However, the study of metabolic patterns often employs different models, such as mouse [14] and humans [40, 41]. For the first time, our research has compared the parallels in GCs metabolism between women and mouse. Importantly, the sampling time points for both species represent biologically equivalent stages of follicular maturity. The collection of mouse samples at 14 h post-hCG (human chorionic gonadotropin) and human samples at about 36 h post-trigger both correspond to the late pre-ovulatory phase, yielding fully expanded COCs containing metaphase II oocytes. We found that the changes in glycine and glucose were identical in women and mouse, while the trend of decreased long-chain fatty acids was similar. This indicates that these metabolites are evolutionarily conserved. These evolutionarily conserved metabolic changes, consistent with reports in other biological systems [33, 42, 43], underscore the fundamental roles of these metabolites in reproductive physiology and the universal metabolic consequences of obesity across species. This cross-species metabolic framework not only advances reproductive metabolism research but also validates the mouse model as a physiologically relevant system for human ovarian metabolic studies.

**Figure 6.**
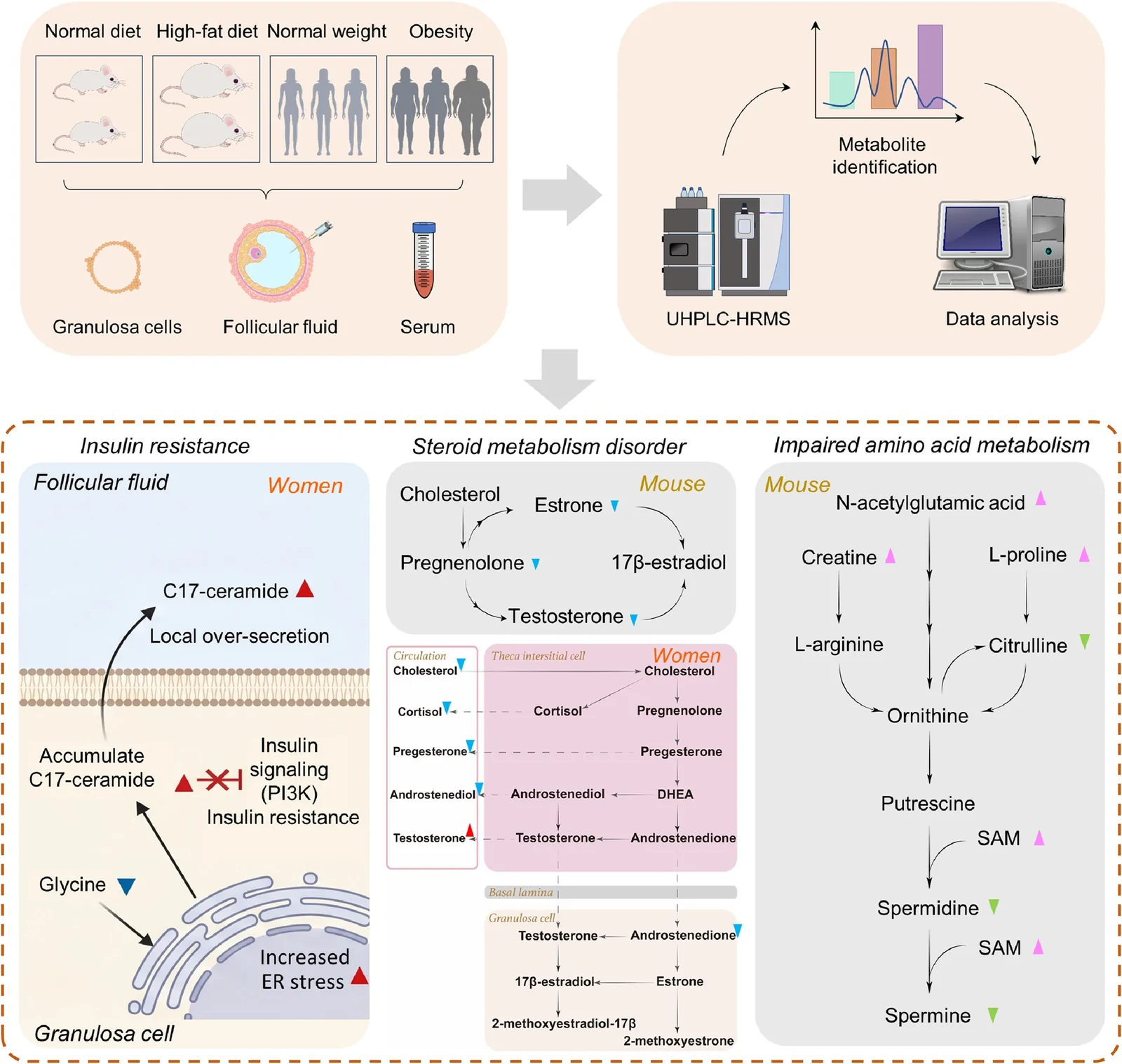
Graphical summary of the cross-species follicular metabolic alterations in obesity. Schematic representation illustrates the parallel metabolic remodeling across mouse and human granulosa cells, as well as distinct compartmental changes in human follicular fluid and serum, highlighting that ceramide accumulation impaired steroidogenesis and altered amino acid polyamine pathway.

Our investigation of metabolic perturbations in the human follicular microenvironment revealed multiple metabolites implicated in IR pathways. Given the ubiquitous expression of insulin receptors in GCs and theca cells, and the established role of insulin in promoting follicular development and luteinizing hormone receptor expression [44], these findings carry significant implications. Notably, we detected elevated ceramide levels in both GCs and FF, consistent with its known role in IR pathogenesis [28]. This observation suggests a sustained IR state within the follicular environment. The heightened IR state in human follicles manifested through multiple metabolic perturbations. GCs exhibited marked glucose depletion, a hallmark feature of IR pathophysiology [45]. This indicates that the energy substrate utilization may transition from carbohydrate metabolism to fatty acid oxidation in GCs, accounting for the observed reduction in long-chain fatty acids. While the precise origin of ceramide accumulation remains unclear, studies have implicated dysregulation in phospholipid metabolism, sphingolipid synthesis, and membrane oxidation pathways [27]. In obese individuals, endoplasmic reticulum (ER) stress is present [46], and a study has shown that glycine can alleviate this stress in oocytes [47]. Notably, we observed downregulation of glycine in both human and mouse GCs, which may render GCs more susceptible to ER stress. It has been demonstrated that ER stress can enhance IR and lipolysis [27], potentially including sphingolipid breakdown, leading to increased ceramide levels, which in turn exacerbate inflammation and IR. Elevated insulin levels can directly stimulate theca cells, promoting androgen secretion [48], which may explain the testosterone elevation amidst global suppression of steroidogenic pathways. Mechanistically, IR in human GCs impairs phosphoinositide 3-kinase (PI3K) signaling, which downregulates aromatase activity and restricts androgen-to-estrogen conversion, thereby exacerbating testosterone accumulation. Furthermore, IR suppresses the steroidogenic acute regulatory protein, hindering mitochondrial cholesterol transport and causing the global reduction of downstream steroids like pregnenolone. Collectively, these findings delineate an obesity-induced metabolic vicious cycle in the follicular microenvironment, wherein IR, ceramide accumulation, and ER stress mutually reinforce one another to disrupt steroidogenic homeostasis and compromise oocyte developmental competence (Fig. 6).

### Research limitations

Despite the comprehensive metabolic profiling presented in our study, several limitations should be acknowledged. First, conclusions are primarily based on high-throughput metabolomics of GCs, FF, and serum. Although this approach identifies significant metabolic alterations, it does not establish direct causality. For example, our proposed links between ceramide accumulation, ER stress, and an insulin-resistant microenvironment are based solely on metabolomic associations. Further functional validation of insulin signaling pathways is required to confirm these hypotheses. Second, the study lacks parallel omic profiling of the oocytes themselves. While GCs provide essential metabolic support through nutrient transfer, the absence of oocyte-specific molecular signatures limits our ability to fully capture intracellular metabolic responses within the gamete. Finally, functional and mechanistic validation in oocyte or GC models has not yet been performed. Future studies integrating targeted functional assays with oocyte multi-omic profiling will be important for clarifying the metabolic mechanisms underlying obesity-associated reproductive disorders.

## Methods

### Research ethics

For mouse studies, all procedures were approved by the Animal Care and Use Committee of Nanjing Medical University, China (Protocol No. IACUC1703017). For human studies, this prospective cohort study was approved by the Institutional Review Board of Center for Reproductive Medicine, Shandong University, China (Approval No. 2021IRB98). Written consent was obtained from each participant.

### Mouse samples

All experiments were approved by the local animal ethical committee and the Animal Care and Use Committee of Nanjing Medical University, China (Protocol No. IACUC-1703017). ICR (Institute of Cancer Research) mice were obtained from the Experimental Animal Center of Nanjing Medical University, China, and house under specific pathogen-free conditions with a 12 h light/dark cycle at constant temperature (22°C ± 2°C) and controlled humidity levels. Three-week-old female mice were randomly divided into two diet groups: one group received HFD (Research Diets, D12492), and the other received ND (Jiangsu Xietong Pharmaceutical Bio-Engineering Co., China, XT101FZ) for 16 weeks.

Female mice were injected intraperitoneally with 5 units of pregnant mare serum gonadotropin, followed by 5 units of hCG 48 h later. Mice were sacrificed by cervical dislocation at 14 h post-hCG injection. For the collection of GCs surrounding MII oocytes, COCs were isolated from the oviduct ampullae and then cumulus masses were digested in M2 medium (Nanjing Luanchuang Co., China, M01-B) containing 1.0 mg/mL hyaluronidase at 37°C for 5 min. Ten sets of samples were meticulously collected from both HFD and ND groups (*n *= 10 for the ND group and *n *= 9 for the HFD group), with about 6.0 × 10^5^ cumulus cells separated from 500 COCs per sample for metabolomics.

### Human samples

This prospective cohort study conducted in 2021, involving 45 women under 35 years old who underwent ICSI (intracytoplasmic sperm injection) and embryo transfer at the Center for Reproductive Medicine Shandong University. Exclusion criteria included age > 35 years, use of donor oocytes, gestational surrogacy, and medical conditions that could affect BMI (e.g. diabetes mellitus, hyperthyroidism, or hypothyroidism). Furthermore, patients diagnosed with polycystic ovary syndrome were strictly excluded to avoid potential confounding effects arising from the coexistence of polycystic ovary syndrome and obesity. The primary infertility diagnosis categories in our cohort were restricted to male factor, tubal factor, and unexplained infertility. We also confirmed that all enrolled individuals had no history of smoking and were not taking any metabolic medications or adhering to specific restrictive diets prior to the study. Written consent was obtained from each participant, and the study was approved by the Institutional Review Board of Center for Reproductive Medicine, Shandong University (2021IRB98).

Follicle size was assessed by ultrasound at oocyte retrieval. Follicular aspirates containing oocytes with surrounding cumulus GCs were collected. After oocyte removal, the remaining material was centrifuged at 3000 *g* for 15 min to isolate GCs. Fluid from the first aspirated follicle was used for measuring hormone and metabolite levels.

Serum was separated for subsequent analysis from blood samples collected from all participants during the early follicular phase at the beginning of the study. Blood samples were collected into serum gel tubes between 8 and 10:30 a.m. after an overnight fast of at least 8 h. The tubes were gently inverted twice and then rested at room temperature for 30 min to allow complete coagulation. Subsequently, the blood was centrifuged at 2750 *g* for 10 min at 15°C to obtain serum, which was stored at −80°C until metabolic analysis.

### Metabolite detection

Metabolome data were collected and analyzed as previously described [16]. Samples were flash-frozen in liquid nitrogen and stored at −80°C. For metabolite extraction, samples were homogenized in 80% methanol/water (*v*/*v*) using an Ultra-Turrax homogenizer, centrifuged at 16,000 *g* for 15 min at 4°C, and the supernatant was stored at −80°C until analysis. Metabolomics analysis was performed using an UPLC Ultimate 3000 system coupled to a Q-Exactive mass spectrometer. Chromatographic separation was achieved on a Hypersil GOLD C18 column at 40°C with a flow rate of 0.4 mL/min. The mobile phase consisted of 0.1% formic acid in acetonitrile (ACN) (A) and 0.1% formic acid in water (B). The gradient elution was as follows: 1% A for 3 min, linearly increased to 99% A at 10 min, maintained for 3 min, and then decreased to 1% A for 2 min. Mass spectrometry was conducted at 70,000 resolutions in both positive and negative modes, with a full-scan range of 70–1050 *m*/*z*.

### Data processing and statistical analysis

Data were processed using TraceFinder v3.1, and metabolites were identified by comparing accurate mass and retention time with commercial standards. Statistical analyses were performed using R (V4.2.0), with Student’s *t*-test for two-group comparisons and OPLS-DA using SIMCA-P (V14.0). Metabolites with VIP >1.00 and *P *< 0.05 were considered significant. Fold change was standardized across species, defined as the ratio of mean metabolite abundance in the OW/OB or HFD group relative to the NW or ND control group. KEGG Mapper (V4.1) and GraphPad Prism 10 were used for data integration and visualization, respectively.

## Supplementary Material

lnag028_Supplementary_Data

## Data Availability

The data produced and analyzed during this study are available from the corresponding authors upon reasonable request.
