# Supplementary material for: Cross-species metabolic profiling of follicular compartments in obesity
Source: Life Med. 2026 Jul 11;5(4):lnag028. doi: 10.1093/lifemedi/lnag028 (PMC13503033; doi:10.1093/lifemedi/lnag028)
Supplement: Supplementary file 1 [file lnag028_supplementary_data.zip › 027_SI.docx]

**Cross-species metabolic profiling of follicular compartments in obesity**

Yiqiu Wu^1,#^, Shiyu Xie^1,#^, Mengyuan Zhu^2,#^, Yu Tian^3^, Hengjie Wang^4^, Shoubin Tang^1^, Minjian Chen^2^, Qiang Wang^5,1,*^, Longsen Han^1,*^, Shuai Zhu^1,*^

^1^State Key Laboratory of Reproductive Medicine and Offspring Health, Nanjing Medical University, Nanjing 211166, China

^2^Key Laboratory of Modern Toxicology of Ministry of Education, School of Public Health, Nanjing Medical University, Nanjing 211166, China

^3^State Key Laboratory of Reproductive Medicine and Offspring Health, Center for Reproductive Medicine, Institute of Women, Children and Reproductive Health, Shandong University, Jinan 250012, China

^4^Nanjing Women and Children’s HealthCare Hospital, Women’s Hospital of Nanjing Medical University, Nanjing 210004, China

^5^Changzhou Maternity and Child Health Care Hospital, Changzhou Medical Center, Nanjing Medical University, Changzhou 213000, China

^#^These authors contributed equally to this work.

^*^Correspondence: [zhus2018@njmu.edu.cn](mailto:zhus2018@njmu.edu.cn) (S.Z.), [qwang2012@njmu.edu.cn](mailto:qwang2012@njmu.edu.cn) (Q.W.), hls@njmu.edu.cn (L.H.)

**Supplemental figure legends**

**Figure S1. Metabolomic profiling of mouse granulosa cells.**

(A) Heatmap visualizing relative abundance of differential metabolites classified by KEGG pathway. (B) Bubble chart of enriched KEGG pathways showing the top 25 enriched metabolite in mice GCs. The vertical dividing line indicates the significance threshold of *P* = 0.05, which corresponds to −log_10_ (*P* value) = 1.3.

**Figure S2. Metabolomic profiling of human granulosa cells, serum and follicular**

**fluid.**

(A–C) OPLS-DA score plot for metabolomic datasets clearly distinguishes GCs (A), serum (B), and FF (C) samples from NW and OW/OB groups. (D) Bubble chart of enriched KEGG pathways showing the top 25 enriched metabolite in woman GCs. The vertical dividing line indicates the significance threshold of *P* = 0.05, which corresponds to −log_10_ (*P* value) = 1.3.
